# The Virtual Summer Research Program: supporting future physician-scientists from underrepresented backgrounds

**DOI:** 10.1017/cts.2022.447

**Published:** 2022-08-22

**Authors:** Briana Macedo, Briana Christophers, Rio Barrere-Cain, Yentli Soto Albrecht, Michael C. Granovetter, Rachit Kumar, Dania Daye, Elizabeth Bhoj, Lawrence Brass, Jose Alexandre Rodrigues

**Affiliations:** 1 Princeton University, Princeton, NJ, USA; 2 American Physician Scientists Association, Westford, MA, USA; 3 Weill Cornell/Rockefeller/Sloan Kettering Tri-Institutional MD-PhD program, New York, NY, USA; 4 University of California, San Francisco, CA, USA; 5 Medical Scientist Training Program of the Perelman School of Medicine at the University of Pennsylvania, Philadelphia, PA, USA; 6 University of Pittsburgh-Carnegie Mellon University Medical Scientist Training Program, Pittsburgh, PA, USA; 7 Harvard Medical School, Cambridge, MA, USA; 8 Children’s Hospital of Philadelphia, Philadelphia, PA, USA; 9 Perelman School of Medicine at the University of Pennsylvania, Philadelphia, PA, USA; 10 Michigan State University College of Osteopathic Medicine, East Lansing, MI, USA

**Keywords:** Diversity, physician-scientists, MD–PhD, DO–PhD, removing barriers to higher education

## Abstract

**Introduction:**

Physician-scientist training programs expect applicants to have had extensive research experience prior to applying. Even at the best of times, this leaves individuals from underserved and underrepresented backgrounds at a competitive disadvantage, especially those remote from major academic centers. The COVID-19 pandemic exacerbated that disadvantage by closing research laboratories and suspending summer research opportunities.

**Methods:**

The Virtual Summer Research Program (VSRP) was designed to combat this shortfall by helping participating students become better informed and better prepared for applying to MD/DO–PhD programs. 156 participants were recruited from historically black colleges and universities and from national organizations for underrepresented trainees. Participants were paired with medical school faculty members and current MD/DO–PhD students from 35 participating institutions. The program lasted for at least 4 weeks and included a short research project, interactive sessions, journal clubs, social events, and attendance at a regional American Physician Scientists Association conference.

**Results:**

In follow-up surveys, participants reported improvements in their science-related skills and in their confidence in becoming a physician-scientist, applying to training programs, and navigating mentorship relationships. A follow-up study completed one year later indicated that participants felt they had benefited from an enhanced skill set, long-term relationships with their mentors, and connections to the physician-scientist community at large.

**Discussion:**

The results suggest that VSRP met its primary goals, which were to provide a diverse group of trainees with mentors, provide skills and resources for MD/DO–PhD application and matriculation and to support the development of longitudinal relationships between VSRP mentees and APSA. VSRP provides an approach that can be applied at an even larger scale when the constraints caused by a global pandemic have lifted.

## Introduction

The need for greater diversity among physicians and physician-scientists has received a great deal of attention. Patients are more likely to adhere to medical advice, have better outcomes, and trust their providers when they share cultural, racial, or gender identity with their physicians [1–3]. Diversity within research teams encourages innovation and is correlated with scientific impact [4,5].

Nevertheless, the NIH 2014 Physician-Scientist Workforce Working Group demonstrated that the pipeline of underrepresented groups into dual degree (MD/DO–PhD) programs is meager and not growing [6]. They reported that only a fraction of applicants and practicing physician-scientists come from backgrounds underrepresented in medicine. Similarly, a recent study revealed that only 8% of MD–PhD matriculants are first in their family to attend college [7]. These numbers fall well short of the demographics of the U.S. population.

Prior research experience is an important part of successfully applying to physician-scientist training programs [8]. Challenges in obtaining research experience contribute to disparities and so does inequitable distribution of information about physician-scientist careers and the process of applying to MD–PhD and DO–PhD training programs. Potential applicants may feel hesitant in committing to pursue an eight-year MD–PhD or DO–PhD program because they perceive themselves as being uncompetitive [9,10].

Coronavirus disease 2019 (COVID-19) transformed the reality of college students in the Spring of 2020, precipitating abrupt transitions to remote learning and financial hardships. A study conducted at Arizona State University found that the pandemic exacerbated socioeconomic inequities. Graduations were delayed, approximately 10% fewer students pursued STEM fields, and 40% of students lost a job offer, internship, or current job [11]. Research opportunities were also interrupted because many research programs were put on hold or canceled, Virtual Summer Research Program (VSRP) was one of a very few programs geared specifically for future MD/DO–PhD trainees that offered programing during the tumultuous 2020 year [12].

The suspension of research programs in the summer of 2020 placed many students at a disadvantage, but especially those from groups underrepresented in medicine and from families that either lacked the experience of navigating the complex medical school admissions process or lacked the resources and social networks required to go where opportunities abound [13]. The American Physician Scientists Association (APSA) is a student-led organization whose mission is to help physician-scientist trainees at all levels realize their educational and professional goals. As part of this mission, APSA developed the VSRP in 2020 to foster productive research partnerships and mentorship relationships during the pandemic.

The goal of VSRP was to conduct a summer research program virtually by pairing mentors, such as principal investigators, postdoctoral scholars, and graduate students, with premedical and pre-graduate trainees, most of whom were from groups underrepresented in medicine by one criterion or another. The program hoped to provide mentees with an appropriate mentor, foster the development of skills and resources for MD/DO–PhD program applications and training, and support longitudinal relationships between mentees and APSA. Our hypothesis was that useful research skills, such as literature review, data analysis and interpretation, and academic writing, can be developed in a virtual research environment and that doing so in a research setting, however briefly, would help participants move ahead with their career preparation [14]. Here, we describe the VSRP, highlight its outcomes, and suggest ways that this model can be adapted in other settings.

## Methods

### Recruitment of students (mentees) and mentors

To recruit students and mentors, the authors reached out to an extensive physician-scientist network: historically black colleges and universities, national organizations of URiM trainees, personal networks, and APSA social media. These networks included physician-scientist trainees of various identities and their respective communities including organizations such as the Medical Student Pride Alliance, The National First Generation and Low-income Medicine Association and Medical Students with Disability and Chronic Illness. A website, flyers, and emails were distributed to these sources which included information about the program and an online application link. 122 faculty mentors were recruited from 35 participating institutions. Half were from the University of Pennsylvania. All mentors who applied were included in the program. Students were enrolled via an online portal, which received 645 applications in the seven days that it was open. Of these 645 applicants, 286 were interested in applying to MD–PhD and DO–PhD programs and had an underrepresented identity. Underrepresented identities include racial/ethnic minorities, first-generation college students, LGBT+, low-income, and disabled. These 286 students then moved on to the second round of the selection program.

### Pairing of students with mentors

A matching algorithm was used to identify potential mentor–mentee pairs. Pairwise scores for every possible mentor–mentee pair were generated from common research areas and interests (with a weight of six) as well as skills that a mentor preferred that a mentee had (with a weight of two). Some mentors requested that their mentees have computer programing experience in R or Python, and some requested skills in scientific literature review and writing. The algorithm identified the top three mentors for each mentee and limited each mentor to a maximum of eight matches. Each mentor was sent the applicant resumes identified by the algorithm and asked to rank these mentees. Students closer to graduation and potential application to MD/DO–PhD programs were prioritized for matching. Based on mentor preferences and spots available, 156 mentor–mentee matches were made.

### Program structure

In addition to the mentored research experience, VSRP included journal club sessions, a weekly interactive series, and social hours. The program required four full-time weeks of research. Research fields included anthropology, computational biology, engineering, epidemiology, genetics, immunology, molecular biology, neuroscience, pharmacology, and psychology. Twelve journal club sessions were led by current MD–PhD and DO–PhD students to introduce participants to novel and innovative scientific research, enhance critical reading skills, analyze academic writings, and learn about effective scientific communication. Four professional development sessions were hosted by leaders within the physician-scientist community. Topics included imposter syndrome, leveraging mentoring, and navigating the physician-scientist path as an underrepresented minority. These sessions sought to complement foundational scientific skills such as data analysis, experimental design, and literature review by adding power skills critical to success [15].

Two virtual social hours were held in partnership with the Student National Medical Association and the First-Generation and Low-Income in Medicine Association. Part of the goal was to connect participants with other medical societies and provide them the opportunity to engage with a community of future and current trainees in the biomedical sciences with whom they may share identities. At the conclusion of the program, participants were expected to present their work at a regional APSA conference to motivate them and provide an additional experience in networking and scientific communication.

To provide an idea of the scope of participant research projects, supplemental table 1 offers a sample of abstract titles from VSRP research projects that were submitted to APSA’s 2020 Southern Regional conference. A sample schedule is provided in supplemental figure 1.

### Program Logistics

Physician-scientist trainees ran VSRP, taking care of duties that included publicizing the program, reviewing applications, matching mentees, and mentors, coordinating programing for professional development sessions and journal club sessions, answering queries via email, and holding office hours. Supplementary table 2 summarizes the tasks and time commitments associated with running this program for 4 weeks.

### Program evaluation

Evaluation of VSRP followed an adapted form of Kirkpatrick’s four-level evaluation model: (1) learner satisfaction and reaction; (2) measures of learning attributed to the program; (3) change to learner’s behavior in the evaluated context, while (4) ultimate positive outcomes on admissions were unable to be evaluated in a short-term manner [16]. Pre-, post-, and follow-up surveys were distributed to participants immediately before, immediately afterwards, and one year after completion of the program. The surveys are included in the Supplement. This study was deemed exempt from full review by the Massachusetts General Hospital Institutional Review Board. Of the 156 participants, 111 (71.2%) completed both the pre- and post-surveys and provided identifying information, which enabled a paired analysis. Qualitative questions in the pre-survey were used for quality improvement purposes. 61 (39.1%) completed the follow-up survey. Demographic information was collected in the pre-survey. In each survey, participants were asked to describe their skill level in several categories as either “Novice, no experience”, “Some familiarity”, “Moderate skills”, or “Very skilled”. These responses were converted to a 4-point Likert scale. Additionally, using a 5-point Likert scale, participants were asked to rate their confidence in becoming a physician-scientist, preparedness in applying to physician-scientist training programs, confidence in navigating mentoring relationships, and contact with mentors.

### Statistics

Statistical analyses were conducted in R v.3.5.2 [16]. For each outcome measure, a general linear model was fit to determine whether a fixed intercept predicted the pairwise difference of pre- and post-survey responses. The alpha criterion was 0.05, and all reported *p*-values are Bonferroni-corrected (multiplied by the total number of tests). Plots were generated using ggplot2 [17–19].

Program matching algorithm, R code, and relevant data files have been posted on github: https://github.com/bmacedo-lgtm/VSRP.

## Results

### Participant demographic and socioeconomic characteristics

**Fig. 1.**
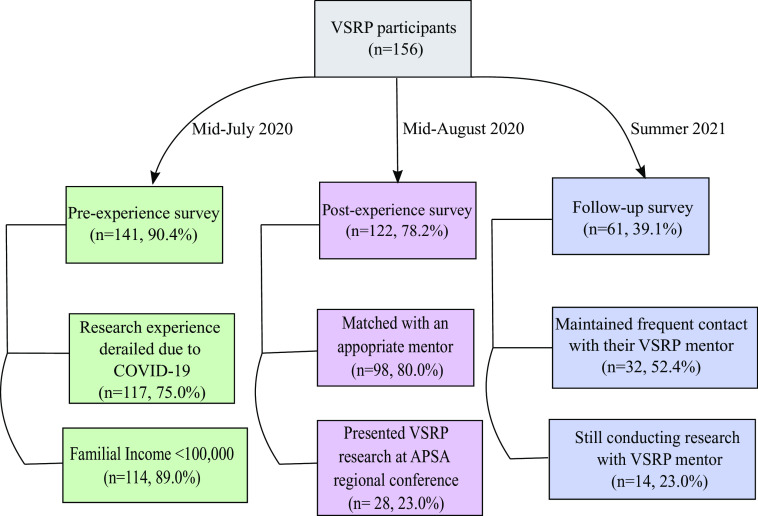
Flow chart demonstrating pre-, post-experience, and follow-up survey participation and selected question results. Virtual Summer Research Program (VSRP) participants (n=156) were asked to participate in three surveys pre-experience (green), post-experience (purple), and follow-up survey (blue), in July 2020, August 2020, and Summer of 2021, respectively. Two questions from each survey are represented, with the number and percentage of respondents who responded in the affirmative. APSA, American Physician Scientists Association.

Supplementary Table 3 summarizes the demographic and socioeconomic characteristics of those who applied and those who participated in the program. 58.7% (n=91) of participants identified as a racial minority, and 25.2% (n=39) identified as Hispanic or Latino. The majority of participants (70.3%, n=109) were female. Many participants identified as LGBTQ+ (16.8%, n=26) or first-generation college students (38.7%, n=60). 89% of the participants who provided data on family income came from families earning <$100,000/year. 34% came from families earning less than $50,000/year. 27% reported that their parents had completed high school or less. Differences between the applicant pool and the participant group reflect a deliberate effort to select participants from underrepresented and underresourced backgrounds. Nearly 80% of participants applied to research opportunities in the summer of 2020 that were canceled due to the COVID-19 pandemic, indicating that VSRP was filling a significant need created by the pandemic (Fig. 1).

### Learner satisfaction and reaction

At the conclusion of the program students reported that they gained valuable analytical skills, developed meaningful relationships with mentors, and grew more confident in their pursuit of a career as a physician-scientist. Many students commented that they developed important scientific skills, and that the program gave them the resources to “excel and grow in scientific knowledge and exploration”. More than 80% (n=98) said they were matched with a compatible mentor; many found their mentor to be inspiring and caring (Fig. 1). One student expressed belonging and support by saying, “I was a real member of the lab family” and “I [gained] a mentor who truly supported me.” Others expressed their increased confidence in applying to an MD-PhD or DO-PhD program “through sitting in lab meetings, APSA journal reviews, and mentorship sessions I was assured that this is the field I am meant to pursue” and describing discussions with their mentor as helping them decide “there is truly no better profession for [them].”

### Measures of learning attributed to the program

**Fig. 2.**
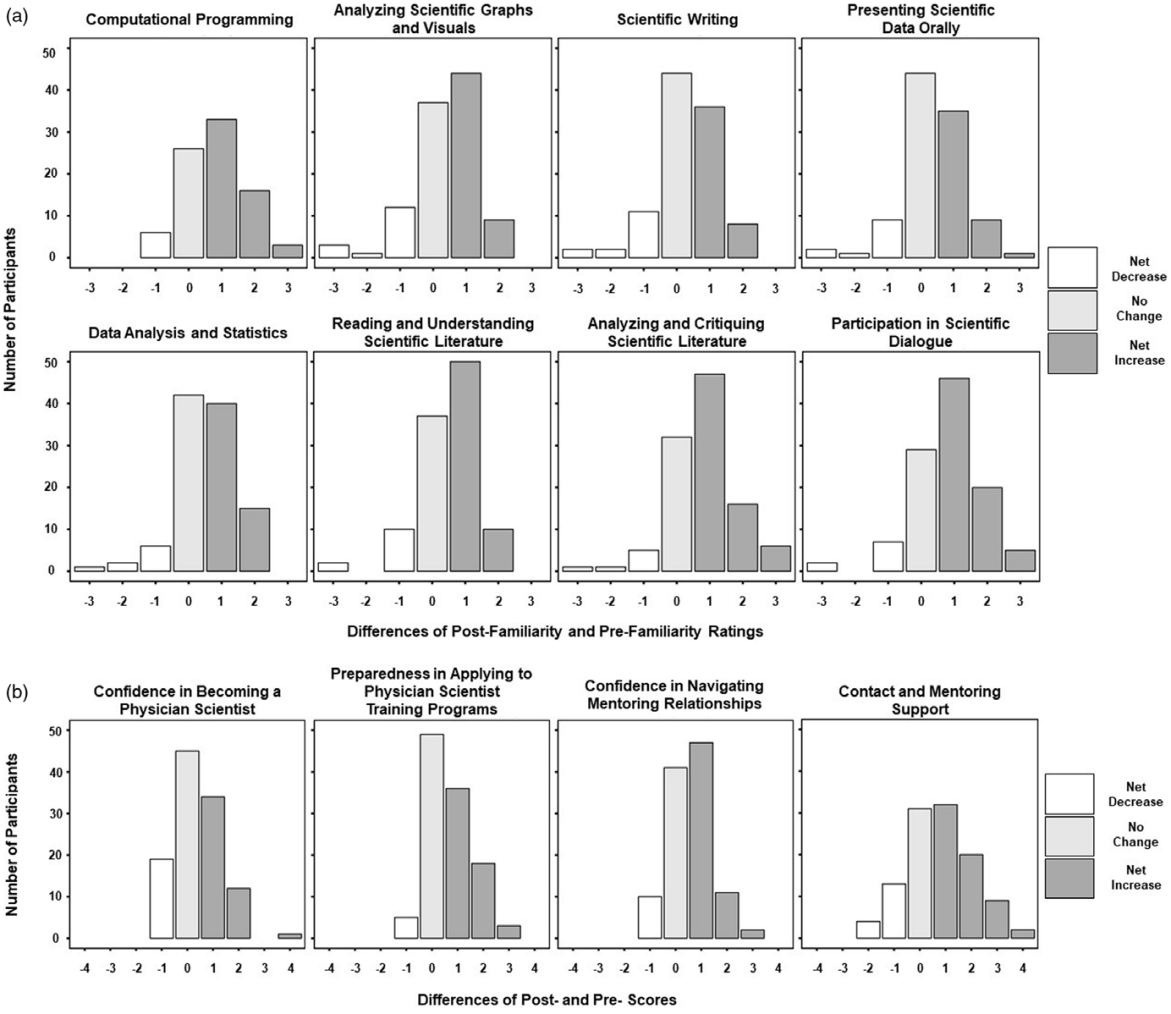
Difference scores on post- vs. pre-VSRP surveyed measures. A: Skill mastery outcomes. B: Outcome measures of Virtual Summer Research Program goals.

Paired surveys completed before and after VSRP asked participants to rate themselves in 12 key areas. They reported a greater sense of mastery in analyzing scientific graphs and visuals, communicating scientific data orally, and analyzing and critiquing scientific literature (Fig. 2A; Table 1). Increased mastery was statistically significant with *p* ≤ 0.01 except for mastery of scientific writing (*p* = 0.03). Participants also reported significantly improved confidence in becoming a physician-scientist, applying to physician-scientist training programs, and navigating mentorship relationships (alpha criterion = 0.01; Fig. 2B; Table 1). 23% (n=42) of participants presented their research at a regional APSA conference and received a certificate of VSRP completion as a result.

**Table 1.** Virtual Summer Research Program outcomes. Survey participants were asked to characterize the level of skill that they felt they had achieved in 12 areas. For each outcome measure, a general linear model was fit to determine whether a fixed intercept predicted the pairwise difference of pre- and post-survey responses (when both were available)

Outcome measure	*t*	df	*p*
Mastery of Computational Programming	7.81	83	1.88 * 10^-10^
Mastery of Analyzing Scientific Graphs and Visuals	3.72	105	3.81 * 10^-3^
Mastery of Scientific Writing	3.15	102	2.54 * 10^-2^
Mastery of Presenting Scientific Data Orally	3.96	100	1.67 * 10^-3^
Mastery of Data Analysis and Statistics	5.90	105	5.31 * 10^-7^
Mastery of Reading and Understanding Scientific Literature	5.63	108	1.75 * 10^-6^
Mastery of Analyzing and Critiquing Scientific Literature	8.15	107	8.89 * 10^-12^
Mastery of Participation in Scientific Dialogue	7.84	108	4.17 * 10^-11^
Confidence in Becoming a Physician-Scientist	4.27	110	4.93 * 10^-4^
Preparedness in Applying to Physician-Scientist Training Programs	8.07	110	1.17 * 10^-11^
Confidence in Navigating Mentorship Relationships	7.19	110	9.82 * 10^-10^
Contact and Mentoring Support	6.25	110	9.38 * 10^-8^

All reported *p*-values are Bonferroni-corrected.

### Change to learner’s behavior in the evaluated context

One year after the conclusion of the program, participants were sent a follow-up survey (Fig. 1). The response rate for this survey was 39% (n=61 of 156). 92% (n=56 of 61) strongly agreed or agreed that they would recommend VSRP. Some reported that VSRP was a “life-changing opportunity” and a “highlight of 2020.” Several stated that VSRP “opened up a lot of new opportunities,” especially since they previously “did not have access” to research; and it enabled them to “build skills to prepare [them] for research.” One participant said that VSRP “gave [them] so much knowledge and the potential for so many connections.”

Those surveyed intended to pursue further graduate studies, with 49% (n=30) of respondents indicating plans to pursue an MD/DO–PhD, 42% (n=26) indicated plans to pursue an MD/DO, and the remaining indicated plans to pursue a Masters (n=4; 6.5%) or a PhD (n=1; 1.6%). Notably, 82% (n=50) said they agreed or strongly agreed that they feel more confident about pursuing a career in biomedical sciences after VSRP, and 62% (n=38) felt that VSRP helped them decide what field to pursue in their career.

The majority of respondents included their work with VSRP on their CV/resume (n=56; 92%) and 71% (n=43) felt that VSRP helped them obtain future research opportunities. The majority of respondents (n=44; 72%) reported that they were conducting research during the summer of 2021 and of those, 23% (n=14) were conducting research with their VSRP mentor from the previous summer. Over half of respondents (n=32; 52%) stated that they were still in frequent contact with their VSRP mentors and another 22% (n=13) said they were in contact for several months after the program conclusion. The majority of the respondents (n=46; 75%) would ask their mentors for a letter of recommendation. One participant said that they were “matched with an amazing mentor that [they] would have never had the chance to meet” outside of VSRP. Others indicated that they continue to research with their mentor and will publish their work as a result of VSRP. Another said that their mentor helped them get a full-time technician job and wrote a letter of recommendation for their MD-PhD applications. One stated that something they “have taken away from the experience is that mentorship (short or long term) is really important and the connections [they] made through VSRP continue to be resources even a year later.”

Almost 60% (n=36) of respondents indicated that they continued to engage with other APSA programing since the end of VSRP 2020. Programming events include virtual interactive sessions where participants learn about applying to MD/DO–PhD programs and career options. Another popular programing event is the APSA undergraduate mentorship program which pairs current MD/DO–PhD trainees with undergraduates.

## Discussion

The goals of the VSRP in 2020 were to replace opportunities that had been curtailed or eliminated by the COVID-19 pandemic; connect participants with enthusiastic mentors, who would help mentees achieve their academic and career goals; provide a research experience which would foster the skills and domain knowledge necessary to obtain future research opportunities; and empower mentees to pursue graduate training - all through serving students who share identities that are underrepresented in science and medicine. The results suggest that VSRP achieved these goals as evaluated by the Kirkpatrick’s four-level evaluation model. Students felt individually supported because of one-on-one relationships with mentors and dedicated program leaders. The majority of survey respondents said that they would recommend the program to others. Even in a four-week program, mentees expressed how the program positively impacted their career trajectory.

The first goal of the program was to provide participants an opportunity to connect with a mentor who could advocate for them and write them letters of evaluation based on their individual strengths. The majority of participants maintained long-term contact with their mentors and a quarter are still working with their mentors. This was an important outcome as four-weeks of research experience in a virtual environment is not independently sufficient to prepare participants for MD/DO–PhD training, application, or matriculation. VSRP facilitated these relationships simply through introduction, and yet this could have a tremendous impact on the trajectories of VSRP participants. The majority of survey respondents will or already have asked their mentors for letters of recommendation to graduate school. Respondents strongly felt that mentorship and networking were key drivers in helping them pursue their goals. Many of the survey open-ended sections included stories about how mentors helped mentees achieve their academic and career goals.

The next goal of VSRP was to provide participants with some of the tools for successful matriculation into MD–PhD or DO–PhD programs. Participants reported significantly improved confidence in becoming a physician-scientist, applying to physician-scientist training programs, and navigating mentorship relationships. This is incredibly important, as confidence in becoming a physician-scientist is a key barrier to applying and matriculating into MD–PhD and DO–PhD programs [9,10]. These results mirror the gains seen in traditional summer research programs, such as the Leadership Alliance [20]. Furthermore, respondents felt that VSRP helped them obtain future research opportunities, because they could include an additional research experience on their resumes, as well as newfound skills they enhanced during their experience. Indeed, half of the respondents of the follow-up survey reported that they were applying to an MD/DO–PhD program and the majority of the remaining were applying to medical school. This suggests that VSRP was successful in partially preparing participants to apply to Medical Science Training Programs.

Finally, VSRP was successful in creating longitudinal relationships between APSA and VSRP participants, with over half of respondents reporting continued interaction with APSA post-program. This data suggests that relationships with APSA, or other professional organizations, can provide a vital longitudinal component to summer research experiences.

VSRP is unique because it is trainee-run, with mentored faculty support including key members of the MD/DO–PhD community. It also has a holistic mentor–mentee matching process. Additionally, the ease of implementation of VSRP means it can serve as a model for future programs that support students from underrepresented backgrounds to find research opportunities at the undergraduate, medical, or graduate student levels. VSRP is flexible to implement because it can operate remotely and with a relatively low cost. Supplementary table two details the tasks needed for program implementation in order to facilitate the model being adopted in other contexts and with other institutions. The remote model is beneficial because it helps connect students from geographically disadvantaged areas to mentors they might otherwise never have met, extending support networks beyond the local communities of participants and better distributing often-geographically concentrated resources. For such a program to succeed, faculty ambassadors are crucial for recruiting passionate mentors at the institutional level, as evidenced by overwhelming success with recruiting mentors at the University of Pennsylvania.

The primary limitation of our analysis is that measures of confidence and analytical skills were done based on individual self-reporting. Furthermore, surveys did not receive full participation for all VSRP mentees, and thus response bias is a possible limitation. Only the student perspective was analyzed, and future work will involve analyzing and improving the mentor experience, and, broadly, the program framework. Finally, given the short timeline of this evaluation, the fourth level of the Kirkpatrick model, such as the impact of VSRP on success in application to MD–PhD or DO–PhD programs, could not be applied [16].

Participant and mentor feedback was incorporated into the design of VSRP 2021. Areas of feedback included: facilitating student camaraderie with additional social hours, increasing the breadth of programing events, and optimizing the matching algorithm. In parallel, more journal club topics were included, and organizers were encouraged to use interactive journal club formats. To enhance and elevate mentor–mentee relationship and acquisition of research skills, the minimum experience length was increased to eight weeks. While increasing program length is an improvement, additional integration of reflection exercises and consistent longitudinal follow-up with these trainees would support long-term retention of these participants within the physician-scientist workforce. The major barrier to continued success and implementation of this program is financial resources including for participant stipends. However, the distributed design of the program provides institutional partners with limited administrative financial commitment and allows for participant community development, networking, and trainee interactions at the national level. While in 2020 the focus of this program was to replace disrupted and canceled programing, in the future VSRP will pivot toward providing first research experiences as these initial experiences are pivotal and can be transformational in their impact on future opportunities.

In summary, the VSRP filled a gap by supporting individuals early in their pursuit of a physician-scientist career, not only in the context of a global pandemic, but in a generalizable way that can reduce inequities in healthcare by diversifying the matriculant pool of MD-PhD/DO-PhD programs.

## Supplementary material

For supplementary material accompanying this paper visit https://doi.org/10.1017/cts.2022.447. click here to view supplementary material
