# Supplementary material for: The Virtual Summer Research Program: supporting future physician-scientists from underrepresented backgrounds
Source: J Clin Transl Sci. 2022 Aug 22;6(1):e120. doi: 10.1017/cts.2022.447 (PMC9549583; doi:10.1017/cts.2022.447)
Supplement: Supplementary file 1 [file S2059866122004472sup001.zip › S2059866122004472sup001.pptx]

## Slide 1
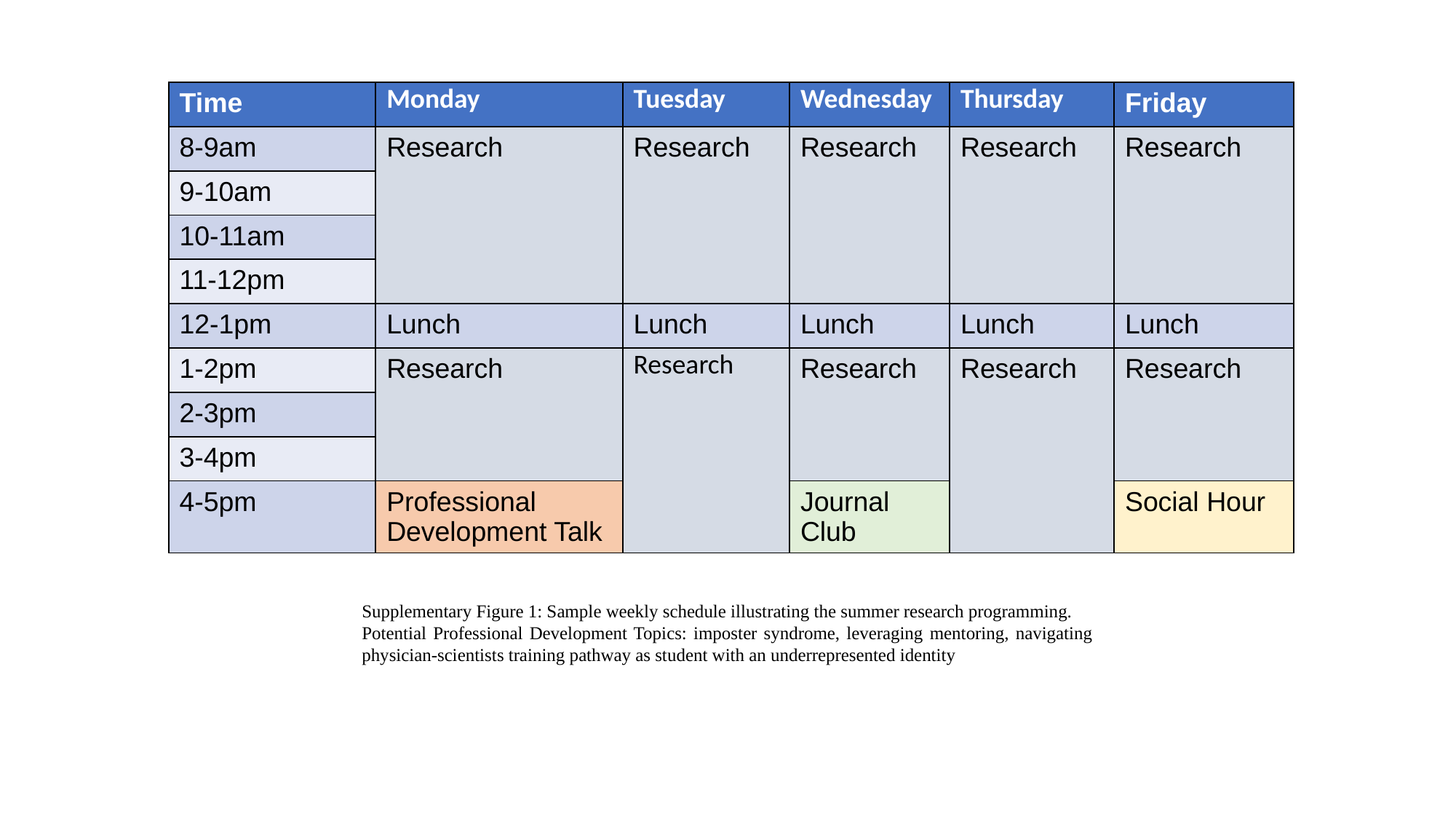

| Time | Monday | Tuesday | Wednesday | Thursday | Friday |
| --- | --- | --- | --- | --- | --- |
| 8-9am | Research | Research | Research | Research | Research |
| 9-10am | | | | | |
| 10-11am | | | | | |
| 11-12pm | | | | | |
| 12-1pm | Lunch | Lunch | Lunch | Lunch | Lunch |
| 1-2pm | Research | Research | Research | Research | Research |
| 2-3pm | | | | | |
| 3-4pm | | | | | |
| 4-5pm | Professional Development Talk | | Journal Club | | Social Hour |
Supplementary Figure 1: Sample weekly schedule illustrating the summer research programming.
Potential Professional Development Topics: imposter syndrome, leveraging mentoring, navigating physician-scientists training pathway as student with an underrepresented identity
