# Supplementary material for: The Virtual Summer Research Program: supporting future physician-scientists from underrepresented backgrounds
Source: J Clin Transl Sci. 2022 Aug 22;6(1):e120. doi: 10.1017/cts.2022.447 (PMC9549583; doi:10.1017/cts.2022.447)
Supplement: Supplementary file 1 [file S2059866122004472sup001.zip › S2059866122004472sup005.docx]

**Supplemental Table 3. Demographic and socioeconomic characteristics VSRP applicants and matched participants.**

| **Race** | **All applicants**  ***n* (%)** | **Matched**  ***n* (%)** |
| --- | --- | --- |
| *American Indian or Alaska Native* | 3 (0.4) | 1 (0.6) |
| *Asian* | 185 (27.7) | 25 (16.1) |
| *Black or African American* | 254 (38.1) | 66 (42.6) |
| *White* | 98 (14.7) | 27 (17.4) |
| *Other* | 85 (12.7) | 14 (9.0) |
| *Multiracial* | 34 (5.1) | 10 (6.5) |
| **Hispanic, Latino, Spanish origin** | 132 (19.8) | 39 (25.2) |
| **Sex** |  |  |
| *Female* | 528 (79.2) | 109 (70.3) |
| *Male* | 126 (18.9) | 36 (23.2) |
| *Non-binary/third gender* | 3 (0.5) | 1 (0.6) |
| *Prefer to not say* | 9 (1.2 ) | 8 (5.2) |
| **LGBTQ+** | 82 (12.3) | 26 (16.8) |
| **Disabled** | 24 (3.6) | 6 (3.9) |
| **Parent education level** |  |  |
| *Some high school, no diploma* | 70 (10.5) | 15 (9.7) |
| *High school graduate or equivalent* | 107 (16.0) | 27 (17.4) |
| *Trade/technical/*  *vocational training* | 13 (1.9) | 1 (0.6) |
| *Some college credit* | 58 (8.7) | 11 (7.1) |
| *Associate’s degree* | 27 (4.0) | 6 (3.9) |
| *Bachelor’s degree* | 148 (22.2) | 46 (29.7) |
| *Master’s degree* | 132 (19.8) | 25 (16.1) |
| *Doctoral degree* | 47 (7.0) | 5 (3.2) |
| *Professional degree* | 31 (4.6) | 7 (4.5) |
| *Prefer to not say* | 34 (5.1) | 12 (7.8) |
| **Family income/year** |  |  |
| *< $25,000* | 126 (18.9) | 30 (19.4) |
| *$25,000 - 49,999* | 157 (25.5) | 38 (24.5) |
| *$50,000 - 74,999* | 113 (16.9) | 23 (14.8) |
| *$75,000 - 99,999* | 77 (11.5) | 23 (14.8) |
| *> $100,000* | 92 (13.8) | 14 (9.0) |
| **Graduating class** |  |  |
| *Graduated before 2020* | 81 (12.1) | 24 (15.5) |
| *2020* | 34 (5.1) | 12 (7.7) |
| *2021* | 166 (24.9) | 29 (18.7) |
| *2022* | 217 (32.5) | 45 (29.0) |
| *2023* | 144 (21.6) | 35 (22.6) |
| *2024* | 17 (2.5) | 2 (1.3) |
| **Planning to apply to dual degree** | 566 (84.9) | 147 (94.8) |
| **Research opportunity derailed due to COVID-19** | 514 (79.7) | 117 (75.0) |
| **Total** | 645 (100.0) | 156 (100.0) |
