# Supplementary material for: The Virtual Summer Research Program: supporting future physician-scientists from underrepresented backgrounds
Source: J Clin Transl Sci. 2022 Aug 22;6(1):e120. doi: 10.1017/cts.2022.447 (PMC9549583; doi:10.1017/cts.2022.447)
Supplement: Supplementary file 1 [file S2059866122004472sup001.zip › S2059866122004472sup004.docx]

| Task | Hours Per Week | Number of Weeks |
| --- | --- | --- |
| Publicizing program to mentees and mentors | 3 | 3 |
| Matching Mentees and Mentors | 15 | 1 |
| Coordinating Programming | 3 | 4 |
| Monitoring Program Email | 3 | 4 |
| Holding Office Hours | 2 | 4 |

Supplementary Table 2: Table summarizing the tasks and time commitments associated with running the virtual summer research program for four weeks.
