# Supplementary material for: The Virtual Summer Research Program: supporting future physician-scientists from underrepresented backgrounds
Source: J Clin Transl Sci. 2022 Aug 22;6(1):e120. doi: 10.1017/cts.2022.447 (PMC9549583; doi:10.1017/cts.2022.447)
Supplement: Supplementary file 1 [file S2059866122004472sup001.zip › S2059866122004472sup003.docx]

**Appendix.** Study questionnaires

**Pre-survey Questionnaire**

1. Email address
2.What is your name?
3. What are you most hoping to achieve during this program?
4. What are you most looking forward to during this program?
5. What do you think will be most challenging about this program?
6. What are you most anxious about regarding this program?
7. How do you think this program will benefit you personally or professionally?
8. Are there resources or opportunities related to this program that you hope the organizers (APSA) could provide?
9. Before this virtual summer research program, please describe your mastery of the following skills.
□ Novice, no experience □ Some familiarity □ Moderate skills □ Very skilled □ N/A

- Computational Programming
- Analyzing scientific graphs and visuals
- Scientific writing
- Presenting scientific data orally
- Data analysis and statistics
- Reading and understanding scientific literature
- Analyzing and critiquing scientific literature
- Participation in scientific dialogue (for example during lab meetings or one-on-one meetings).

10. On a scale of 1 to 5 (5 being the most confident), how would you rate your confidence in becoming a physician scientist?
□ 1 □ 2 □ 3 □ 4 □ 5
11.On a scale of 1 to 5 (5 being the most prepared), how would you rate your preparedness in applying to physician scientist training programs?
□ 1 □ 2 □ 3 □ 4 □ 5
12. On a scale of 1 to 5 (5 being the most confident) how would you rate your confidence in navigating mentorship relationships?
□ 1 □ 2 □ 3 □ 4 □ 5
13. On a scale of 1 to 5 (5 being the most support) how much contact and mentoring support do you have from individuals in professions related to science and medicine?
□ 1 □ 2 □ 3 □ 4 □ 5
14. Did you use the Resource Packet?
□ Yes □ No
15. If so, how helpful was it (5 being the most helpful)?
□ 1 □ 2 □ 3 □ 4 □ 5

**Post-Experience Survey Questionnaire**

1. Email address

2. Name

3. Graduation Year

4. Undergraduate institution

5. In what ways can the matching process improve?

6. Please estimate your total hours spent with VSRP activities (research, meetings, preparing, programming)

7. The matching process enabled me to match with an appropriate mentor.

□ Strongly Agree □ Agree □ Neutral □ Disagree □ Strongly Disagree

8. I communicated with my direct mentor

□ Daily

□ 2-3 Times Weekly

□ Once a week

□ < 4 times throughout the entire experience

9. I met with the principal investigator of the lab at least twice during this experience

□ Yes □ No

10. How many Journal Clubs did you attend

□ 1 □ 2 □ 3 □ 4 □ 5+

11. The journal club sessions taught me to review, read and constructively analyze literature

□ Strongly Agree □ Agree □ Neutral □ Disagree □ Strongly Disagree

12. In what ways can the journal clubs improve?

13. Did you find the Physician-Scientist mentorship series useful?

□ Strongly Agree □ Agree □ Neutral □ Disagree □ Strongly Disagree

□ Other: _____________

14. How many physician-scientist mentoring series sessions did you attend?

□ Imposter Syndrome in the Mentor-Mentee Relationship with Dr. Tiffany N. Brown, Ph.D. and Dr. Oana Tomescu, MD PhD

□ Leveraging Mentoring with Dr. Ruth Gotian, EdD, MS

□ Achieving Balance: Navigating Challenges and Opportunities as an Underrepresented Minority and Physician Scientist with Dr. Meghan Kirksey,MD., PhD.

□ My Journey as a Physician-Scientist: From Snake Tongues to Human Brains with Dr. Elizabeth Bhoj, MD., PhD.

15. Please rank each of the physician-scientist mentorship series with your perception of their impact.

□ Extremely Useful □ Somewhat Useful □ Neutral □ Somewhat not useful □ Not useful

16. How could the physician-scientist mentorship series be improved?

17. Did you attend any of the Social Hours?

□ Student National Medical Association (SNMA) MD/PhD Social Hour

□ FGLIMed (first generation, low income) Social Hour

□ Did not attend either session

18. How could the Social Hours be improved?

19. Did you feel a sense of camaraderie with your fellow students in the program?

□ Strongly Agree □ Agree □ Neutral □ Disagree □ Strongly Disagree

20. Do you have suggestions on how the program could have increased that sense of camaraderie?

21. After this virtual summer research program, please describe your mastery of the following skills.

□ Novice, no experience

□ Some familiarity

□ Moderate skills

□ Very skilled

□ N/A (Did not attend)

- Computational Programming
- Analyzing scientific graphs and visuals
- Scientific writing
- Presenting scientific data orally
- Data analysis and statistics
- Reading and understanding scientific literature
- Analyzing and critiquing scientific literature
- Participation in scientific dialogue (for example during lab meetings or one-on-one meetings).

22. On a scale of 1 to 5 (5 being the most confident), how would you rate your confidence in becoming a physician scientist?

□ 1□ 2 □ 3 □ 4 □ 5

23. On a scale of 1 to 5 (5 being the most prepared), how would you rate your preparedness in applying to physician scientist training programs?

□ 1 □ 2 □ 3 □ 4 □ 5

24. On a scale of 1 to 5 (5 being the most confident) how would you rate your confidence in navigating mentorship relationships?

□ 1 □ 2 □ 3 □ 4 □ 5

25. On a scale of 1 to 5 (5 being the most support) how much contact and mentoring support do you have from individuals in professions related to science and medicine?

□ 1 □ 2 □ 3 □ 4 □ 5

26. Would you like to join the American Physician Scientist Association (APSA) mentorship program? Select YES if you have not already signed up. □ Yes □ No

27. Would you like to attend virtual interactive sessions throughout the year that are aimed at preparing students for MD-DO/PhDprogram applications? □ Yes □ No

28. Will you be continuing research with your mentor past this summer?

□ Yes □ No

29. Any additional feedback

30. Were you provided a stipend?

□ Not provided

□ Yes

31. How much were you provided?

□ $1000

□ $500

□ Other:

32. Who provided this funding?

33. This amount sustained me for the four weeks and enabled me to participate in my virtual experience

□ Strongly Agree □ Agree □ Neutral □ Disagree □ Strongly Disagree

34. General feedback about the funding process or amount

**Longitudinal Survey Questionnaire**

​​1. Email

2.Name

3.Do you intend to pursue a postgraduate degree?

□ MD/DO-PhD □ MD/DO □ PhD □ Masters □ No □ Not sure

4.Which cycle do you intend on applying to graduate school?

□ 2021 □ 2022 □ 2023 □ 2024 □ 2025+ □ Unsure □ Already applied

5. Are you pursuing research this summer?

□ Yes □ No

​​6. If yes, where are you doing research?

□ With a professor at my home university

□ With my VSRP mentor from last summer

□ Another summer research program

□ Fellowship

□ Research technician/assistant job

□ Not applicable

7. Have you engaged in any American Physician Scientists Association (APSA) programming since the conclusion of VSRP?

□ Yes

□ No

8. If yes, which programming?

□ Interactive sessions

□ Mentorship program

□ Undergraduate local chapter

□ Regional events

□ Not applicable

□ Other:

9. How long did you remain in contact with your VSRP mentor after the conclusion of the program

□ Days

□ Weeks

□ Months

□ I am still in contact with my mentor

10. Would you ask your VSRP mentor for a letter of recommendation?

□ Yes, definitely

□ Yes, probably

□ Probably not

□ Definitely not

11. Did you include VSRP on your resume/CV?

□ Yes

□ No

12. Rate the following statement on a scale of 1-5 (5 being strongly agree): Creating a poster presentation for an APSA regional conference prepared me for future poster presentations

13. Rate the following statement on a scale of 1-5 (5 being strongly agree): VSRP helped me decide what field I want to pursue as in my career?

14. Rate the following statement on a scale of 1-5 (5 being strongly agree): I feel more confident about pursuing a career in biomedical sciences after VSRP.

15. Rate the following statement on a scale of 1-5 (5 being strongly agree): Participating in VSRP helped me obtain future research opportunities

16. Please describe your mastery of the following skills.

□ Novice, no experience □ Some familiarity □ Moderate skills □ Very skilled □ N/A (Did not attend)

- Computational Programming
- Analyzing scientific graphs and visuals
- Scientific writing
- Presenting scientific data orally
- Data analysis and statistics
- Reading and understanding scientific literature
- Analyzing and critiquing scientific literature
- Participation in scientific dialogue (for example during lab meetings or one-on-one meetings).

17. On a scale of 1 to 5 (5 being the most support) how much contact and mentoring support do you have from individuals in professions related to science and medicine?

18. On a scale of 1 to 5 (5 being the most confident) how would you rate your confidence in navigating mentorship relationships?

19. On a scale of 1 to 5 (5 being the most confident), how would you rate your confidence in becoming a physician scientist?

20. On a scale of 1 to 5 (5 being the most prepared), how would you rate your preparedness in applying to physician scientist training programs?

21. Have you ever used the APSA resource packet after completing VSRP?

□ Yes, often

□ Yes, sometimes

□ Yes, rarely

□ Never

22. Rate the following statement on a scale of 1-5 (5 being strongly agree): I would recommend VSRP to others.

23. Was there anything you wish you could have learned or experienced through VSRP, but you did not?

24. Do you have any other comments about VSRP?
